# Carcinoma Buccal Mucosa Underlying a Giant Cutaneous Horn: A Case Report and Review of the Literature

**DOI:** 10.1155/2014/518372

**Published:** 2014-07-15

**Authors:** Sunil Kumar, Priyank Bijalwan, Sunil K. Saini

**Affiliations:** ^1^Department of Surgical Oncology, IRCH, All India Institute of Medical Sciences, New Delhi 110029, India; ^2^Division of Surgical Oncology, Cancer Research Institute, HIHT, Jollygrant 248140, India

## Abstract

Cutaneous horn is a conical, dense, and hyperkeratotic protrusion that often appears similar to the horn of an animal. Giant cutaneous horns are rare; no incidence or prevalence has been reported. The significance of cutaneous horns is that they occur in association with, or as a response to, a wide variety of underlying benign, premalignant, and malignant cutaneous diseases. A case of giant cutaneous horn of left oral commissure along with carcinoma left buccal mucosa is reported here as an extremely rare oral/perioral pathology.

## 1. Introduction

Cutaneous horn is a relatively uncommon lesion, also known by the Latin name “Cornu cutaneum.” It is conical hyperkeratotic projection above the surface of the skin that often resembles the horn of an animal. It may be straight or curved and twisted and vary from a few millimeters to several centimeters in length [1, 2]. The earliest documented case of cutaneous horn was that of an elderly Welsh woman in London who was displayed commercially as an anomaly of nature in 1588 [3]. There were several other accounts of cutaneous horns in the sixteenth and seventeenth centuries, including those described by Danish anatomist Thomas Bartholin in 1670. The London surgeons Everard Home and his brother-in-law John Hunter are generally credited with the characterization of cutaneous horns as a medical disorder in the late eighteenth century [4]. Cutaneous horns most frequently occur in sites that are exposed to actinic radiation or burns and, hence, are typically found on upper parts of the face. Although there are multiple reports of cutaneous horns, giant cutaneous horns are much rarer. Over 60% of the lesions are benign; however malignant or premalignant lesions might be associated with it. In this paper we describe a unique case of a 45-year-old man with a giant cutaneous horn at left oral commissure with underlying verrucous carcinoma of left buccal mucosa. This is a very rare location for such lesions.

## 2. Case Presentation

**Figure 1:**
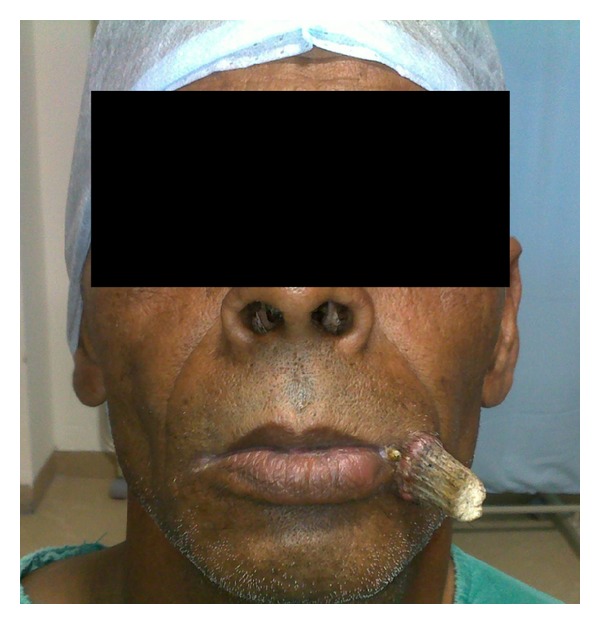
Cutaneous horn at the left angle of mouth.

A 45-year-old male presented with complain of a slowly growing conical asymptomatic lesion at the left angle of mouth. The lesion had been present for the past 2 years and was gradually increasing in size despite the patient having cut off its tip several times. Patient also complained of a lesion over left buccal mucosa which was present for the past two months and was gradually progressive. History of cigarette smoking for the past 30 years was present. Past medical history was insignificant. On examination, a hard, grey coloured, conical keratinized lesion measuring 3 × 1 cm was present over the left angle of mouth (Figure 1).

**Figure 2:**
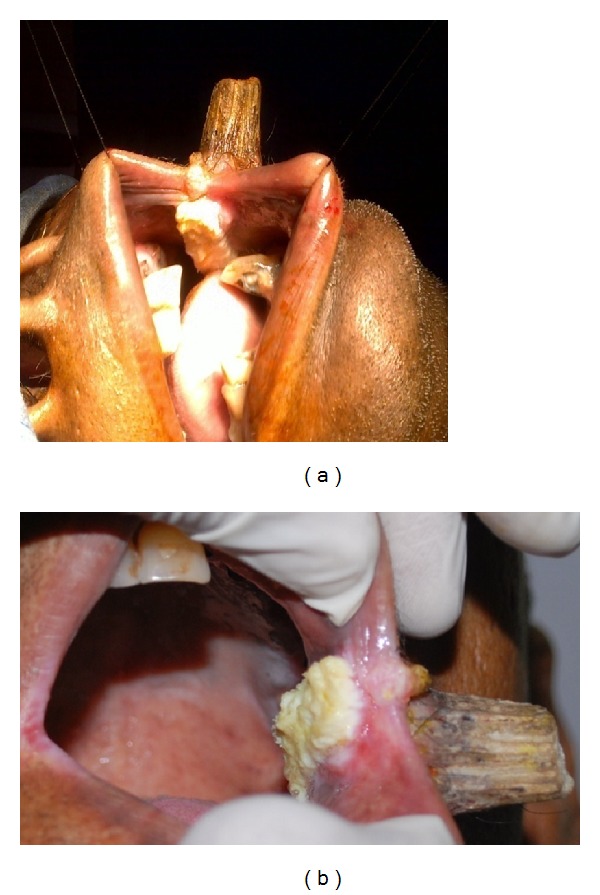
(a) Growth in the buccal mucosa underlying the cutaneous horn. (b) Close-up view.

Oral examination showed a 4 × 1 cm velvety proliferative growth over the left buccal mucosa extending posteriorly from the left oral commissure (Figures 2(a) and 2(b)).

Neck examination was suggestive of levels I and II cervical lymphadenopathy on the left side. Preoperative work-up included routine blood investigations and X-ray chest which were within normal limits. FNAC from neck node was inconclusive. Punch biopsy of the mucosal lesion was suspicious for verrucous carcinoma. Under general anaesthesia and nasotracheal intubation patient underwent wide local excision of the buccal mucosa growth taking 1 cm margin along with excision of the conical cutaneous lesion with supraomohyoid neck dissection of the left cervical nodes. Primary closure of the defect was done in 3 layers. Postoperative period was uneventful and patient was discharged on postoperative day 5.

**Figure 3:**
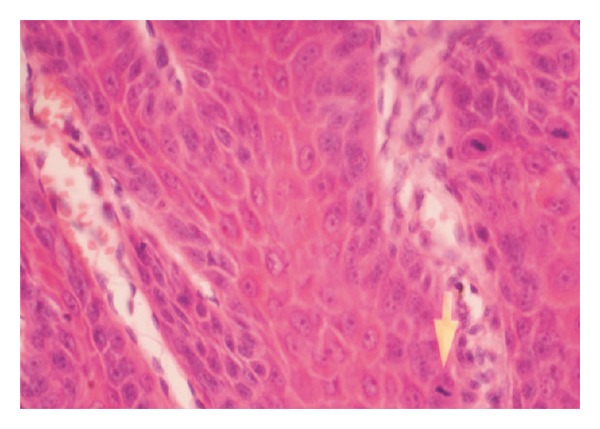
Photomicrograph of verrucous carcinoma of buccal mucosa with an arrow at the areas of atypical keratinocytes.

**Figure 4:**
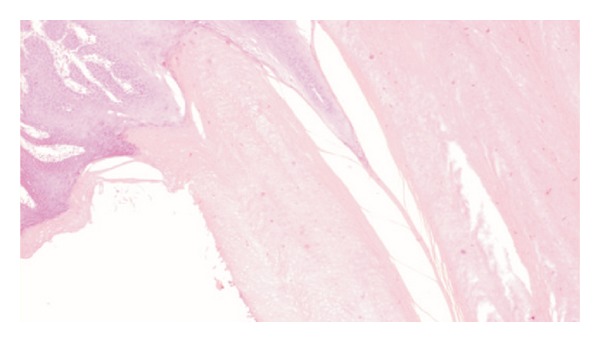
Photomicrograph showing the hyperkeratosis adjacent to the areas of verrucous carcinoma.

Histopathological examination of the surgical specimen showed features consistent with verrucous carcinoma of buccal mucosa with hyperkeratosis underlying the horn (Figures 3 and 4). All neck nodes were free of tumor. No clinical relapses were detected after 2 years of follow-up.

## 3. Discussion 

The term “cutaneous horn” is a morphologic designation referring to unusually cohesive keratinized material and it is not a true pathologic diagnosis. Cutaneous horns though grossly similar to horns in animals are histologically quite different from them. The animal horns are composed of superficial hyperkeratotic epidermis, dermis, and centrally positioned bone. No such axially positioned well-formed bone is observed in the gigantic human horns. On the other hand, no cystic structures lined by trichilemmal type epithelium are seen in those of the true animal horns [5]. Majority of the cases occur on areas that are exposed to sunlight. Forearm, cartilaginous portion of the ear, leg, and back of hands may also be involved [6]. The angle of mouth is a comparatively rare site for cutaneous horn. Giant cutaneous horns of lip are comparatively rarer and malignancies associated with them are even more uncommon. On review of the literature, 9 cases in the past have been reported to have cutaneous horn over the lip and three of these had an associated squamous cell carcinoma at its base [7–9]. The important issue is not the horn itself which is dead keratin, but rather the underlying condition, which may be benign (seborrheic keratosis, viral warts, histiocytoma, inverted follicular keratosis, verrucous epidermal nevus, and molluscum contagiosum), premalignant (solar keratosis, arsenical keratosis, and Bowen's disease), or malignant (squamous cell carcinoma, rarely, basal cell carcinoma, metastatic renal carcinoma, granular cell tumor, sebaceous carcinoma, or Kaposi's sarcoma) [10]. Malignancy has been described in 16–20% of cases, with squamous cell carcinoma being the most common type. A study by Bart et al. reported 44% of the patients having underlying malignancy [11]. In a study involving 643 cutaneous horns reported by Yu et al., 39% of cutaneous horns were derived from malignant or premalignant epidermal malignancy [12]. Features associated with malignant or premalignant histopathology at the base of a cutaneous horn include advanced age, male gender, sun exposed lesion site, and geometry of the lesion. Lesions with a wide base or a low height-to-base ratio are more likely to show malignant base pathology. The association of oropharyngeal cancer with various subtypes of human papilloma virus (HPV) has been described in the literature. There are also reports of HPV being associated with cutaneous horns but a vast majority of them have been found in association with warts. Routine evaluation for HPV infection in patients with oral cancers is not followed at our institution but on revisiting the histopathology slides we could not find any evidence of HPV infection in this patient.

Though cutaneous horn can be removed by simple detachment and cauterization of the base, a full thickness wide local excision with an adequate margin should be obtained for histopathological analysis, keeping in mind the frequent association of malignant/premalignant changes at the base. Cryosurgery is not advocated for the treatment of giant cutaneous horns as it does not ensure full thickness excision of the tumor and also it is not appropriate for the treatment of squamous cell carcinomas.

## 4. Conclusion

Cutaneous horns are predominantly benign lesions; however possibility of nearly one third of them harboring malignant or premalignant skin lesions should be kept in mind. Full thickness excision with margin should be the treatment of choice to enable detailed pathological examination of the underlying tissue.
